# Distinct Effects of Dexamethasone on Human Natural Killer Cell Responses Dependent on Cytokines

**DOI:** 10.3389/fimmu.2017.00432

**Published:** 2017-04-13

**Authors:** David J. Morgan, Daniel M. Davis

**Affiliations:** ^1^Manchester Collaborative Centre for Inflammation Research, University of Manchester, Manchester, UK

**Keywords:** glucocorticoids, natural killer cells, cellular activation, dexamethasone, cytokines

## Abstract

Glucocorticoids (GCs) have long been known to be immune suppressive and synthetic variants are widely used in the treatment of inflammatory disorders. Here, we report that, while suppressing the initial production of interferon-γ (IFN-γ), the synthetic GC dexamethasone (Dex) enhances the proliferation and survival of natural killer (NK) cells stimulated with interleukin-2 (IL-2) + interleukin-12 (IL-12). Inhibition of mTOR complex 1 by rapamycin revealed the immunosuppressive activity of Dex was independent from the effect of enhancing NK cell proliferation. In the presence of IL-2 + IL-12, Dex also increased the percentage of NK cells that were CD16^+^ and DNAM1^bright^, increased the level of expression of CD94 or NKG2A, and improved mitochondrial function of NK cells. Moreover, NK cells treated with cytokines IL-2 and IL-12 + Dex, followed by a 7-day rest, displayed an increased IFN-γ response upon restimulation. Thus, there is a dichotomic effect of GCs on NK cell function dependent on the local cytokine milieu; the NK cell effector response is initially suppressed, but, dependent on the cytokines present, Dex can also augment the proliferation, survival, and reactivity of human NK cells in a secondary recall response.

## Introduction

Natural killer (NK) cells are lymphocytes that play a critical role in the innate immune response through the secretion of inflammatory cytokines and lysis of viral-infected or neoplastic cells (1–5). Accordingly, humans lacking NK cells or displaying a low NK cell activity show an increased susceptibility to infection and cancer development (6, 7). Further evidence for the importance of NK cells is that improved patient outcome often correlates with high numbers of intratumoral NK cells (8).

Natural killer cell effector functions are triggered through germline-encoded activating receptors, such as NK group member D (NKG2D) and CD16, and cytokine receptors, such as the interleukin-2 receptor, IL-12R, and IL-15R (9–13). Although NK cells are part of the innate immune system on account of using germline-encoded receptors for their activation, recent studies have demonstrated that NK cells also possess attributes associated with adaptive immunity, such as clonal expansion and the generation of long-lived memory cells (3, 14–18). In addition to antigen-specific memory, NK cells also exhibit a cytokine-induced long-lasting memory-like response (16, 19). Understanding NK cell regulation is clinically important in, for example, establishing protocols for generating long-lived functional NK cells for cancer treatments (20).

Glucocorticoids (GCs) are a class of stress-induced steroid hormones that act through the GC receptor (GR), affecting a diverse range of physiological processes including energy metabolism, cell fate, and immunity. GCs possess potent anti-inflammatory properties, at least in part by inhibiting the effector functions of immune cells, such as T cells, macrophages, and NK cells, resulting in their widespread use in the treatment of inflammatory disorders and in cancer (21–28). However, there is also some evidence to indicate that GCs could have a stimulatory effect on immune cells, including macrophages and T cells (25, 29, 30). Stimulation of the human NK cell line, NK92, with interleukin-2 (IL-2) or interleukin-12 (IL-12), following a 5-day culture with GCs, resulted in enhanced IL-6 or interferon-γ (IFN-γ), respectively (31). Furthermore, hydrocortisone with either IL-2 or interleukin-15 (IL-15) had a synergistic effect on peripheral blood-derived CD56^+^ cells to increase expansion (32).

Here, we establish that the synthetic GC, dexamethasone (Dex), greatly enhances the proliferation of primary human NK (pNK) cells stimulated with IL-2 + IL-12. In addition, Dex protects pNK cells stimulated with IL-2 + IL-12 from cytokine-induced cell death. Despite exerting a positive influence on pNK cell proliferation and survival, GCs still displayed potent suppressive effects on IFN-γ production initially. Thus, dependent on the local cytokine stimulation, Dex may suppress IFN-γ production initially but augment IFN-γ secretion in a secondary recall response.

## Materials and Methods

### Cell Culture and Maintenance

Primary human NK cells were obtained from healthy donor peripheral blood as previously described (33, 34). In brief, peripheral blood mononuclear cells were isolated by density gradient centrifugation (Ficoll-Paque Plus; Amersham Pharmacia Biotech) and pNK cells were isolated using negative magnetic selection (NK cell isolation kit; Miltenyi Biotec), resulting in a purity of >98% (CD56^+^CD3^−^CD19^−^). Freshly isolated cells were cultured at 10^6^ cells/ml in clone medium: Dulbecco’s modified Eagle’s medium supplemented with 30% Nutrient Mixture F-12 HAM, 10% human serum, 1 mM non-essential amino acids, 1 mM sodium pyruvate (all from Sigma-Aldrich), 2mM l-glutamine, 1 mM penicillin, 1 mM streptomycin, and 50 µM 2-mercaptoethanol (all from Gibco). pNK cells were expanded with 200 U/ml human recombinant IL-2 (Roche) and were rested for 6 days prior to use, unless stated otherwise. For experimental procedures, pNK cells were washed and resuspended in clone medium supplemented with 10% charcoal dextran stripped fetal calf serum (sFCS; Gibco), to remove endogenous GCs from medium.

### Cytokine Stimulation of pNK Cells

Following expansion with IL-2 for 6 days, pNK cells were resuspended at 0.5 × 10^6^ cells/ml, in clone medium supplemented with 10% sFCS, instead of human serum. pNK cells were pretreated with 100 nM dexamethasone (Dex, used as a 1 mM stock solution; Sigma-Aldrich) or dimethylsulfoxide (DMSO; Sigma-Aldrich), as a vehicle control for 1 h. pNK cells were also pretreated with 1 µM Ru486 (a GR antagonist; used as a 20 mM stock solution; Sigma-Aldrich), where indicated. Human recombinant IL-2 (200 U/ml), human recombinant IL-12 (10 ng/ml; Peprotech), human recombinant IL-15 (5 ng/ml; Peprotech), and human recombinant interleukin-18 (IL-18; 100 ng/ml; MBL International) were then added as indicated, and the pNK cells were incubated for either 18 h or 5 days. Concentrations used were comparable to those previously used to stimulate pNK cells (19, 20). Where indicated, 10 nM rapamycin (used as a 2.5 mM stock solution; Calbiochem) was also added, with DMSO used as a vehicle control. A concentration of 10 nM was selected as it has previously been shown to prevent IL-2 induced expression of granzyme B in pNK cells (35).

### Quantitative RT-PCR (qRT-PCR)

Cells were treated and stimulated with cytokines as described and then incubated for either 18 h or 5 days. RNA was extracted and qRT-PCR was performed as previously described (34). Target gene expression was assessed using the following primer pairs: glyceraldehyde-3-phosphate dehydrogenase, forward primer, 5′-GAAGGGTGAAGGTCGGAGT-3′, reverse primer, 5′-CATGGGTGGAATCATATTGGAA-3′; IFN-γ, forward primer, 5′-AAAAATAATGCAGAGCCAAATTG-3′, reverse primer, 5′-TAGCTGCTGGCGACAGTTCA-3′; tumor necrosis factor-α (TNF-α), forward primer, 5′-TCTTCTCGAACCCCGAGTGA-3′, reverse primer, 5′-CCTCTGATGGCACCACCAG-3′; glucocorticoid-induced leucine zipper (GILZ), forward primer, 5′-TGTGGATGAGGGATGAACAA-3′, reverse primer, 5′-ACCCGCTACAGACAAGCTTT-3′; and FK506-binding protein 5 (FKBP5), forward primer, 5′-TGTCTCCCACGTGTGTATTAT-3′, reverse primer, 5′-TTTGCTCAGAACCACTCACAC-3′.

### Enzyme-Linked Immunosorbent Assays (ELISAs)

Primary human NK cells were treated and stimulated as described above and then incubated for 18 h or 5 days. Supernatants were collected and IFN-γ secretion was analyzed by ELISA, as previously described (34).

### Flow Cytometry

Primary human NK cells were treated and stimulated as described and incubated for 5 days. Stimulated cells were washed and resuspended in flow buffer (0.5% fetal bovine serum/PBS) before cell surface staining was performed at 4°C, with fluorophore-conjugated antibodies against the following proteins: CD56 (clone HCD56, Biolegend), CD16 (clone 3G8, Biolegend), NKG2D (clone 1D11, BD Biosciences), CD57 (clone HNK-1, Biolegend), NKG2A (clone 131411, R&D Systems), CD94 (clone DX22, Biolegend), and DNAM1 (clone TX25, Biolegend). Isotype-matched control antibodies conjugated with the appropriate fluorophores were used in parallel (mouse IgG1, κ isotype control, clone MOPC-21, Biolegend; mouse IgM, κ isotype control, clone MM-30, Biolegend; mouse IgG1, κ isotype control, clone MOPC-21, BD Biosciences; mouse IgG2A isotype control, clone 20102, R&D Systems). Staining was performed with appropriate combination of fluorophores. Cell viability was assessed using a Live/Dead stain (Zombie NIR™ Fixable Viability kit, Biolegend). Cells were then washed and resuspended in flow buffer before fixation with 2% paraformaldehyde/0.25% fetal bovine serum/PBS at 4°C. Cells were analyzed by flow cytometry, and data analysis was performed using FlowJo_v10 software (Tree Star). Cell debris was excluded, and cells were gated on live single CD56^+^ pNK cells.

### Flow Cytometric Analysis of Proliferation and Cell Death

To assess proliferation and cell death, fresh or rested pNK cells were stained with CellTraceCFSE (carboxyfluorescein diacetate succinimidyl ester; 0.5 µM; Life Technologies), according to the manufacturer’s instructions. Cells were then either pretreated and stimulated as outlined above and incubated for 5 days or immediately fixed with 2% paraformaldehyde/PBS to measure the initial level of CFSE staining (T0). Following incubation, surface staining was performed with a fluorophore-conjugated antibody against CD56 (clone HCD56, Biolegend) and a Live/Dead stain (Zombie NIR™ Fixable Viability kit, Biolegend). Cells were fixed with 2% paraformaldehyde/0.25% fetal bovine serum/PBS and analyzed by flow cytometry. Data analysis was performed using FlowJo_v10 software (Tree Star). To determine cell proliferation, cell debris was excluded and cells were gated on live single CD56^+^ pNK cells. For assessment of cell death, cellular debris was removed and cell death was determined by plotting forward scatter against a Live/Dead stain, Zombie Dead Cell Marker.

### Flow Cytometric Analysis of Mitochondrial Quality

Primary human NK cells were treated and stimulated as described, and then incubated for 5 days. pNK cells were then stained with a fluorophore-conjugated antibody against CD56 and a Live/Dead stain, and then incubated with various dyes (all from Life Technologies), in clone medium, as follows: MitoTracker Green (100 nM) for 1 h at 37°C to measure mitochondrial mass, tetramethylrhodamine methyl ester (100 nM) for 1 h at 37°C to measure mitochondrial membrane potential, and MitoSOX red (5 µM) for 15 min at 37°C to measure mitochondrial-associated reactive oxygen species (ROS). Cells were assessed by flow cytometry and data analyzed using FlowJo software. Cell debris was excluded, and cells were gated on live single CD56^+^ pNK cells.

### Analysis of NK Cell Secondary Responses

Primary human NK cells were plated at 0.5 × 10^6^ cells/ml in clone medium supplemented with 10% sFCS instead of human serum and treated with 100 nM Dex (or vehicle control) for 1 h. Cells were then initially stimulated using IL-2 (200 U/ml) + IL-12 (10 ng/ml), or IL-12 (10 ng/ml) + IL-15 (5 ng/ml) + IL-18 (100 ng/ml), or with IL-2 (200 U/ml) alone as a control condition for 5 days. Following stimulation, supernatants were retained and evaluated for IFN-γ release by ELISA to confirm stimulation. Cells were washed three times and cultured in clone medium supplemented with IL-2 (200 U/ml) to support survival, with medium being replaced every 2–3 days. After 7 days, cells were harvested, resuspended, and plated at either equal densities or using the entire population present for each condition following rest *in vitro*. Cells were then restimulated with IL-12 (10 ng/ml) + IL-18 (100 ng/ml) for 18 h, with IL-2 (200 U/ml) used as an unstimulated control, and IFN-γ release was assessed by ELISA.

### Statistical Analysis

Data are presented as mean values ±SD. Statistical differences were evaluated with a two-tailed unpaired Student’s *t*-test or one-way analysis of variance and Tukey posttest, as indicated. *p* < 0.05 was considered significant. Graphs were produced, and statistical comparisons were performed with GraphPad Prism.

## Results

### Dexamethasone Augments the Proliferation and Survival of NK Cells in the Presence of IL-2 + IL-12

Glucocorticoids are well established to inhibit multiple outcomes of NK cell activation, including proliferation (27), but GCs have also been reported to have an immunostimulatory effect (32). Here, we set out to examine the effect of Dex on NK cell proliferation in response to cytokine stimulation. For this, pNK cells were first labeled with CFSE and pretreated with Dex for 1 h. Cells were then stimulated with cytokines, either individually or in combination. Following 5 days in culture, proliferation, indicated by a dilution of CFSE as cells divide, was quantified by flow cytometry. A small reduction in CFSE staining is evident as some level of fluorescence is lost over 5-day culture; proliferation of NK cells is indicated by subsequent dilutions beyond this.

**Figure 1:**
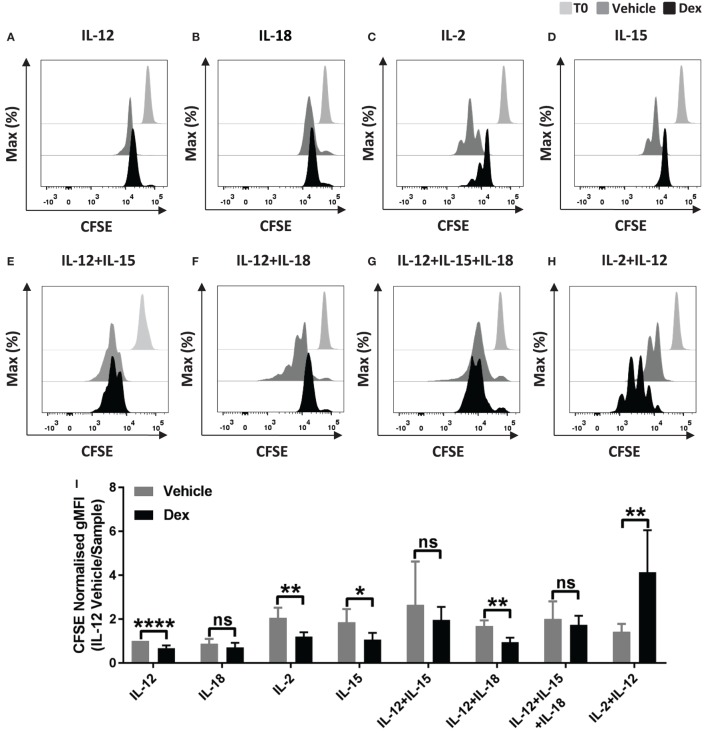

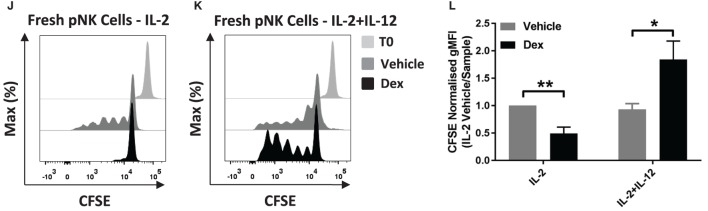
**Glucocorticoids can enhance the proliferation of primary human NK (pNK) cells depending on the cytokine environment**. **(A–I)** pNK cells and **(J–K)** freshly isolated pNK cells were first labeled with CFSE (0.5 µM) and then treated with DMSO, as a vehicle control, or 100 nM dexamethasone (Dex) for 1 h. pNK cells were then stimulated with either **(A)** interleukin-12 (IL-12) (10 ng/ml), **(B)** interleukin-18 (IL-18) (100 ng/ml), **(C,J)** IL-2 (200 U/ml), **(D)** interleukin-15 (IL-15) (5 ng/ml), **(E)** IL-12 + IL-15, **(F)** IL-12 + IL-18, **(G)** IL-12 + IL-15 + IL-18, or **(H,K)** IL-2 + IL-12, for 5 days. Proliferation was assessed by flow cytometry. Representative histograms (gated on live CD56^+^ pNK cells) for **(A)** six, **(B)** three, **(C,D)** six, **(E)** three, **(F)** four, **(G)** four, **(H)** six, or **(J,K)** three independent experiments are shown. **(I)** Graph (mean ± SD) depicts the quantification of geometric mean fluorescence intensity (gMFI) for **(A–H)**, normalized to IL-12 vehicle. **(L)** Graph (mean ± SD) depicts the quantification of gMFI for **(J,K)**, normalized to cells treated with IL-2/vehicle control. Samples are compared by unpaired, two-tailed Student’s *t*-test (*p < 0.05; **p < 0.005; ****p < 0.0001; ns, not significant). T0 denotes initial CFSE stain.

Representative histograms indicate that culture with IL-12 or IL-18 did not support proliferation of pNK cells (Figures 1A,B). Culture with different combinations of cytokines, with or without Dex, clearly induced pNK proliferation to varying degrees (Figures 1C–I). The addition of GCs significantly reduced pNK cell proliferation stimulated by IL-2 or IL-15 alone or IL-12 + IL-18 (Figures 1C,D,F,I). No effect of GC was seen when cells where stimulated with IL-12 + IL-15, or IL-12 + IL-15 + IL-18 (Figures 1E,F,I). However, an unexpected increase in NK cell proliferation was seen for cells pretreated with Dex and stimulated with IL-2 + IL-12 (Figures 1H,I).

In these experiments, pNK cells were expanded in IL-2 for 6 days prior to use, a commonly used method for increasing cell numbers *in vitro*, but also known to alter the nature of NK cells. Thus, we next assessed the effect of Dex on the proliferation of freshly isolated pNK cells. For freshly isolated pNK cells, Dex again inhibited the IL-2 induced proliferation while increasing proliferation stimulated with IL-2 + IL-12 (Figures 1J–L).

**Figure 2:**
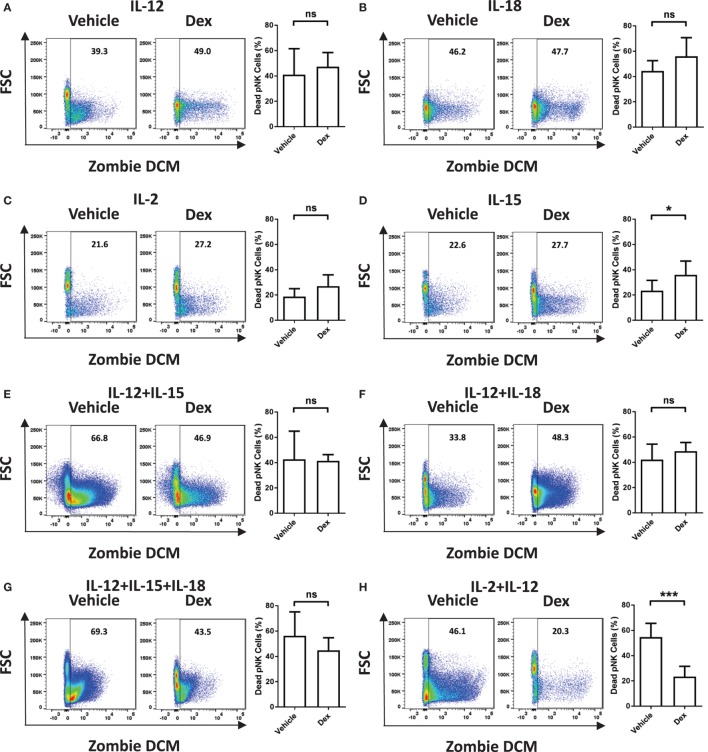

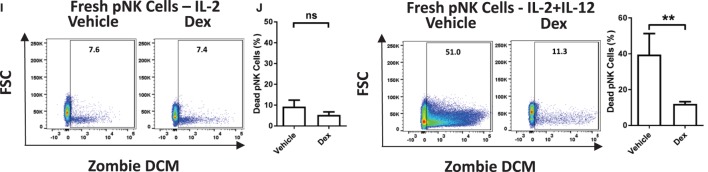
**Glucocorticoids prevent cytokine-induced cell death of primary human NK (pNK) cells**. **(A–H)** pNK cells and **(I–J)** freshly isolated pNK cells were treated with a vehicle control or Dex (100 nM) for 1 h, then cultured for 5 days with either **(A)** interleukin-12 (IL-12) (10 ng/ml), **(B)** interleukin-18 (IL-18) (100 ng/ml), **(C,I)** IL-2 (200 U/ml), **(D)** interleukin-15 (IL-15) (5 ng/ml), **(E)** IL-12 + IL-15, **(F)** IL-12 + IL-18, **(G)** IL-12 + IL-15 + IL-18, or **(H,J)** IL-2 + IL-12. Natural killer cell survival was assessed by flow cytometry. Left panels display representative dot plots of forward scatter (FSC) against Live/Dead stain, Zombie Dead Cell Marker (DCM) for pNK cells. Right panels show percentage of dead pNK cells (mean ± SD) for **(A)** six, **(B)** three, **(C,D)** seven, **(E)** three, **(F,G)** four, **(H)** six, or **(I,J)** four independent experiments. Samples are compared by unpaired, two-tailed Student’s *t*-test (*p < 0.05; **p < 0.005; ***p < 0.0005; ns, not significant).

Immune cells are known to be susceptible to GC-induced cell death. Therefore, we next assessed the survival of Dex-treated pNK cells in response to cytokine stimulation. The proportion of dead pNK cells following 5 days of culture in a variety of cytokines, with and without Dex, was assessed by flow cytometry (Figure 2; gating strategy in Figure S1 in Supplementary Material). Stimulation with either IL-12 (41 ± 8% dead cells; Figure 2A) or IL-18 (44 ± 5% dead cells; Figure 2B) resulted in a decrease in cell survival, when compared to IL-2 (19 ± 2% dead cells; Figure 2C). Unsurprisingly, given both IL-2 and IL-15 are known to support pNK cell survival, stimulation with IL-15 (23 ± 3% dead cells Figure 2D) had no effect on pNK cell viability when compared to IL-2. However, stimulation with a range of cytokine combinations—IL-12 + IL-15 (43 ± 13% dead cells; Figure 2E), IL-12 + IL-18 (42 ± 6% dead cells; Figure 2F), IL12 + IL-15 + IL-18 (56 ± 9% dead cells; Figure 2G), or IL-2 + IL-12 (55 ± 4% dead cells; Figure 2H)—resulted in a significant decrease in pNK cell survival. Dex only had an effect on pNK cell survival following incubation with IL-15 (Figure 2D), where it led to a slight induction of pNK cell death, or IL-2 + IL-12 (Figure 2H), suppressing cytokine-induced cell death, significantly enhancing survival. Cell viability was also assessed for freshly isolated pNK cells (Figures 2I,J). Similarly, the stimulation of freshly isolated pNK cells with IL-2 + IL-12 (40 ± 12% dead cells; Figure 2J) led to significant cytokine-induced cell death when compared to IL-2 (9 ± 2% dead cells; Figure 2I). The addition of Dex suppressed cell death induced by IL-2 + IL-12, significantly enhancing the survival of fresh pNK cells (Figures 2I,J).

Next, we used the GR antagonist Ru486 to test whether or not the effect of Dex on proliferation and survival was mediated by the GR. To verify that a 1 µM dose of Ru486 was sufficient to suppress GC action in pNK cells, we evaluated the expression of two GC-target genes, GILZ and FKBP5. As expected, Ru486 blocked the Dex-induced expression of GILZ and FKBP5 (Figures S2A,B in Supplementary Material). pNK cell proliferation was clearly induced by culture with IL-2 or IL-2 + IL-12, as demonstrated by representative histograms (Figure S2C in Supplementary Material) and the addition of Ru486 abolished the enhanced proliferation and survival induced by Dex in pNK cells stimulated with IL-2 + IL-12 (Figures S2C–F in Supplementary Material). Taken together, these results establish that GCs can enhance the proliferation and survival of pNK cells stimulated by IL-2 + IL-12, in a manner dependent on the GR.

It is well established that mitochondrial function is inherently linked to cell survival, with dysfunction resulting in cell death (36). Therefore, we next evaluated the effect of Dex on the mitochondrial function of pNK cells stimulated with IL-2 + IL-12 for 5 days. Mitochondrial mass remained unchanged in cells stimulated with IL-2 alone or with IL-2 + IL-12, with Dex having little if any effect (Figures S3A,B in Supplementary Material). However, compared to IL-2, stimulation with IL-2 + IL-12 led to a decrease in mitochondrial membrane potential (Figures S3C,D in Supplementary Material) and an increase in mitochondrial-associated ROS (Figures S3E,F in Supplementary Material). Importantly, both of these changes to mitochondrial function were abrogated by the presence of Dex. Thus, the enhanced survival of pNK cells induced by GCs (Figure 2H) correlates with changes in NK cell mitochondria.

### Dexamethasone Suppresses NK Cell IFN-γ Production Independent of Its Effect on the Proliferation of NK Cells

**Figure 3:**
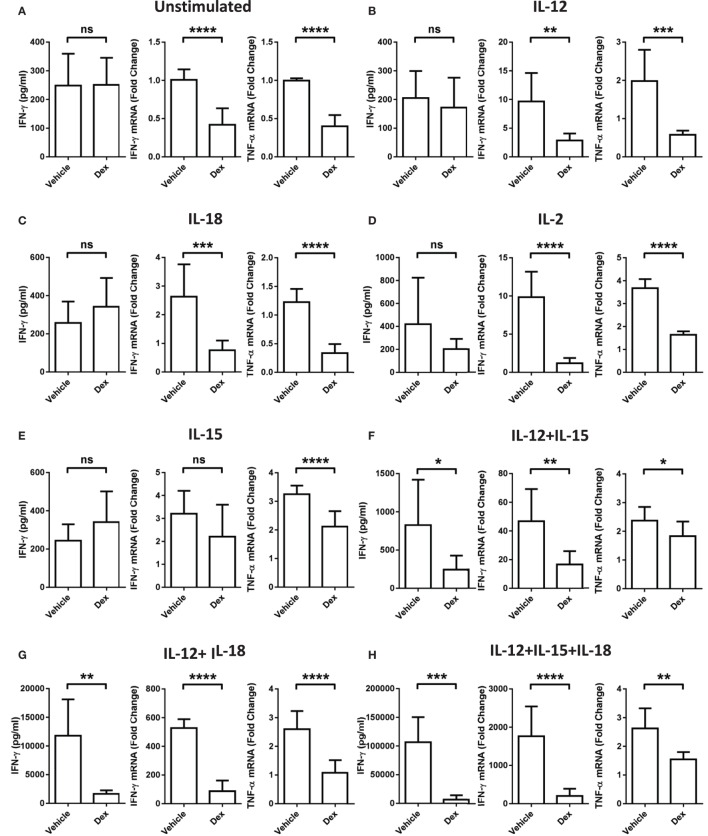

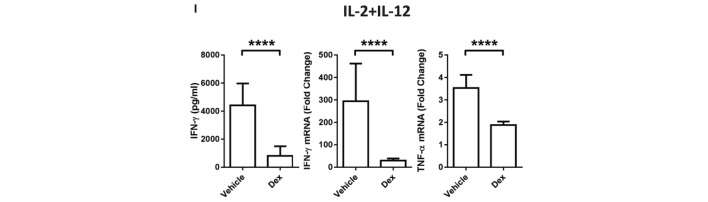
**Glucocorticoid treatment suppresses interferon-γ (IFN-γ) production and secretion from natural killer (NK) cells**. Primary human NK cells were pretreated with a vehicle control or Dex (100 nM) for 1 h, then either, remained **(A)** unstimulated, or were stimulated with **(B)** interleukin-12 (IL-12) (10 ng/ml), **(C)** interleukin-18 (IL-18) (100 ng/ml), **(D)** IL-2 (200 U/ml), **(E)** interleukin-15 (IL-15) (5 ng/ml), **(F)** IL-12 + IL-15, **(G)** IL-12 + IL-18, **(H)** IL-12 + IL-15 + IL-18, or **(I)** IL-2 + IL-12 for 18 h. IFN-γ release was measured by enzyme-linked immunosorbent assay. Graphs (mean ± SD) combine data for **(A)** five, **(B)** five, **(C)** three, **(D,E)** four, **(F–H)** three, or **(I)** four independent experiments. Production of IFN-γ and tumor necrosis factor-α (TNF-α) mRNA was analyzed by RT-qPCR. Data are normalized to the housekeeping gene glyceraldehyde-3-phosphate dehydrogenase and displayed as a fold change over the unstimulated vehicle control (mean ± SD), for **(A)** nine, **(B–D)** three, **(E)** four, **(F–H)** three, or **(I)** eight independent experiments for IFN-γ and **(A)** four, **(B–D)** three, **(E)** four, **(F–H)** three, or **(I)** four independent experiments for TNF-α production. Samples are compared by unpaired, two-tailed Student’s *t*-test (*p < 0.005; **p < 0.005; ***p < 0.0005; ****p < 0.0001; ns, not significant).

While it is well established that GCs are able to exert potent immune suppressive effects, these data indicate a stimulatory effect of Dex on pNK cells dependent on the cytokines present. To clarify whether or not the conditions that favor enhanced proliferation and survival by Dex still lead to suppression of other effector functions, the production of IFN-γ and TNF-α mRNA and the extent of IFN-γ secretion was assessed. After 18 h in the presence of individual cytokines, IL-12, IL-18, IL-2, or IL-15, there was no induction of IFN-γ secretion compared to pNK cells incubated in cytokine-free media (hereon referred to as the unstimulated control) (Figures 3A–E). However, when cytokines were given in combination, there was a dramatic induction of IFN-γ secretion, which was suppressed by Dex (Figures 3F–I). The production of both IFN-γ and TNF-α mRNA was triggered to varying degrees by the stimulation of pNK cells with a variety of different individual cytokines and cytokine combinations (Figure 3). Dex dampened IFN-γ and TNF-α gene expression in all conditions (Figure 3), except for the production of IFN-γ induced by IL-15 (Figure 3D). This immunosuppressive effect of Dex on IFN-γ production and secretion was abolished by Ru486 (Figures S2G,H in Supplementary Material), confirming the involvement of GR.

Since proliferation and survival of pNK cells was significantly enhanced by Dex following a 5-day culture period (Figures 1H and 2H), we also assessed whether the inhibitory action of GCs was retained throughout this period. Indeed, Dex continued to suppress the secretion (Figure S4A in Supplementary Material) and production (Figure S4B in Supplementary Material) of IFN-γ in pNK cells over a 5-day culture period. Thus, despite enhancing pNK cell proliferation and survival, GCs still mediate potent immunosuppressive effects in terms of effector cytokine production.

Recently, mTOR has been revealed to have a central role in NK cell proliferation and activation (35). Therefore, to evaluate whether there is a link between the effects of GCs on proliferation and the cytolytic activity of pNK cells, we used rapamycin, a pharmacological inhibitor of mTOR complex 1. Previous work has demonstrated that rapamycin augments Dex-induced expression of GR target genes (37). Consistent with this, in pNK cells stimulated with IL-2 + IL-12, rapamycin enhanced the Dex-induced expression of GILZ (Figure S4C in Supplementary Material), confirming 10 nM to be sufficient to regulate GR function in pNK cells.

**Figure 4:**
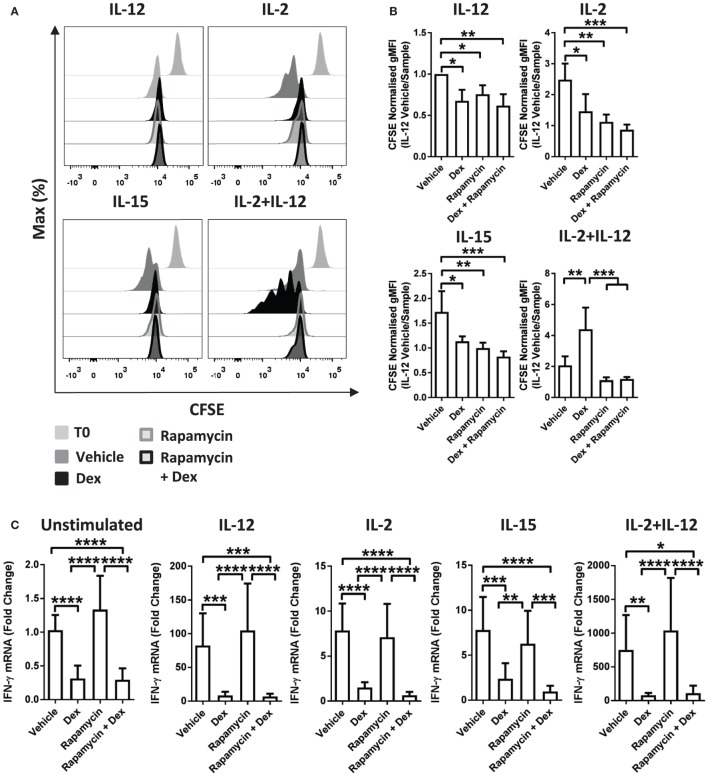

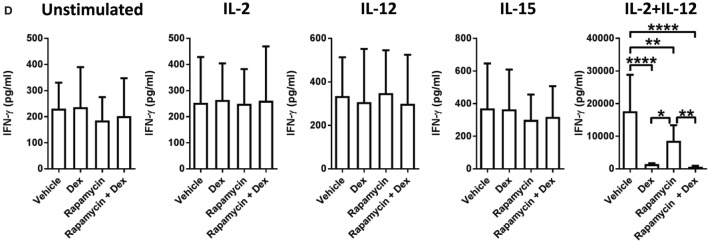
**Immunosuppressive effects of glucocorticoids are not dependent on enhanced proliferation**. **(A,B)** Primary human NK (pNK) cells were first labeled with CFSE (0.5 µM) and then treated with DMSO, as a vehicle control, or Dex (100 nM) for 1 h. Natural killer (NK) cells were then treated with rapamycin (10 nM), as indicated, and cultured for 5 days with either interleukin-12 (IL-12) (10 ng/ml), interleukin-2 (IL-2) (200 U/ml), interleukin-15 (IL-15) (5 ng/ml), or IL-2 + IL-12. Proliferation was assessed by flow cytometry. **(A)** Representative histograms (gated on live CD56^+^ pNK cells) and **(B)** quantification of geometric mean fluorescence intensity, normalized to IL-12 vehicle, are shown. Graphs (mean ± SD) depict four independent experiments. **(C,D)** pNK cells pretreated for 1 h with a vehicle control or Dex (100 nM) were treated with rapamycin (10 nM) and left unstimulated or stimulated with either IL-12 (10 ng/ml), IL-2 (200 U/ml), IL-15, or IL-2 + IL-12 for 18 h. **(C)** Interferon-γ (IFN-γ) gene expression was determined by RT-qPCR. Data are normalized to glyceraldehyde-3-phosphate dehydrogenase and displayed as a fold change over the unstimulated vehicle control (mean ± SD), for five (unstimulated), five (IL-12), four (IL-2), four (IL-15), or four (IL-2 + IL-12) independent experiments. **(D)** IFN-γ secretion was quantified by enzyme-linked immunosorbent assay. Graphs (mean ± SD) depict six (unstimulated), four (IL-12), four (IL-2), three (IL-15), or five (IL-2 + IL-12) independent experiments. Samples are compared by one-way analysis of variance (*p < 0.05; **p < 0.005; ***p < 0.0005; ****p < 0.0001). For simplicity, only significant results are labeled. T0 denotes initial CFSE stain.

The presence of rapamycin inhibited proliferation of pNK cells induced by IL-2 or IL-15. Furthermore, rapamycin completely abolished the enhanced proliferation induced by Dex in IL-2 + IL-12 stimulated cells (Figures 4A,B). However, rapamycin had no influence on the suppressive effect of Dex on cytokine production or secretion: expression of IFN-γ remained unchanged in pNK cells stimulated with IL-2 + IL-12 in the presence of rapamycin and Dex compared to Dex alone (Figures 4C,D). Thus, while rapamycin can abolish the Dex-induced enhancement of pNK cell proliferation, it has no effect on how GCs regulate the production of effector cytokines. This demonstrates that the immediate action of GCs in suppressing IFN-γ can be uncoupled from the effects of GCs on proliferation.

### Dexamethasone Alters the Surface Expression of Several Activating NK Cell Receptors

**Figure 5:**
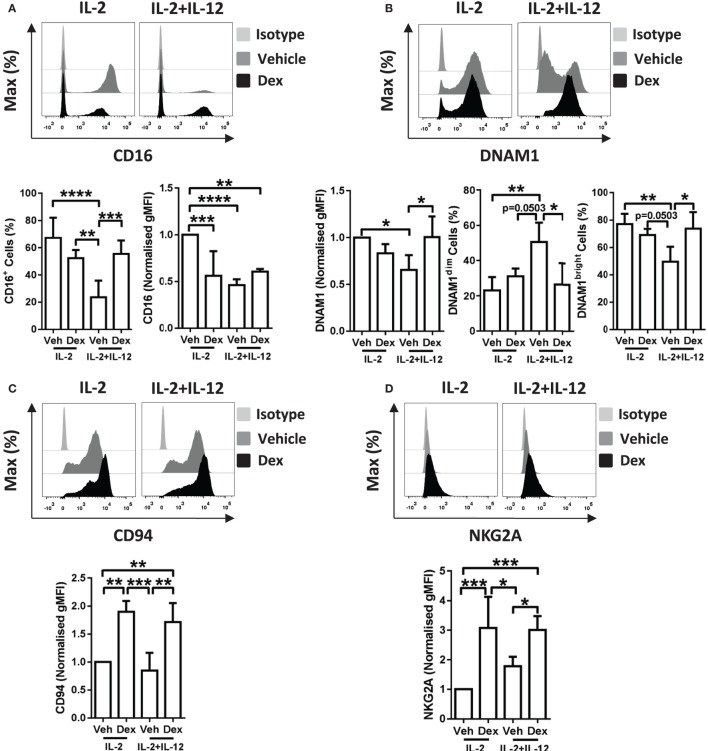
**Dexamethasone alters receptor expression on cytokine stimulated natural killer (NK) cells**. Primary human NK cells pretreated with Dex (100 nM) for 1 h, were cultured for 5 days with either interleukin-2 (IL-2) (200 U/ml) or IL-2 (200 U/ml) + interleukin-12 (IL-12) (10 ng/ml). Cells were assessed for **(A)** CD16, **(B)** DNAM1, **(C)** CD94, and **(D)** NKG2A by flow cytometry. Representative histograms are shown. For panel **(A)**, CD16 graphs depict the percentage of cells positive for CD16, and the geometric mean fluorescence intensity (gMFI) of the positive populations, normalized to cells treated with IL-2 + DMSO (vehicle control). Graphs for **(B)** DNAM1, **(C)** CD94, and **(D)** NKG2A display the gMFI, normalized to cells treated with IL-2 + DMSO (vehicle control). **(B)** The proportion of DNAM1^dim^ and DNAM1^bright^ cells is also shown. Data (mean ± SD) combine **(A)** six, **(B)** four, **(C)** four, or **(D)** five independent experiments. Samples are compared by one-way analysis of variance (*p < 0.05; **p < 0.005; ***p < 0.0005; ****p < 0.0001). For simplicity, only significant results are labeled.

We have demonstrated that the addition of GCs leads to enhanced proliferation of pNK cells stimulated with IL-2 + IL-12, a characteristic typically considered immune-stimulatory, while suppressing the production of IFN-γ. To further understand these observations, we next evaluated changes in the expression of receptors linked with NK cell activation and production of IFN-γ (9, 11, 38–41). These were assessed after stimulation for 5 days with IL-2 + IL-12, with or without Dex (Figure 5; Figure S5 in Supplementary Material). The level of expression of NKG2D was unaltered by pretreatment with Dex in pNK cells stimulated with IL-2 or IL-2 + IL-12 (Figure S5A in Supplementary Material). Similarly, the level of expression and percentage of cells positive for CD57 was unaltered by Dex (Figure S5B in Supplementary Material). However, the expression of CD16, DNAM1, CD94, and NKG2A were affected (Figure 5).

In detail, stimulation of pNK cells with IL-2 + IL-12 resulted in a lower percentage of cells positive for CD16 when compared to treatment with IL-2 (Figure 5A, bottom left panel). This reduction in the percentage of cells positive for CD16 was prevented when pNK cells were pretreated with Dex prior to stimulation with IL-2 + IL-12 (Figure 5A, bottom left panel). Moreover, when compared to pNK cells stimulated with IL-2, the level of expression of CD16 was significantly reduced by pretreatment with Dex or stimulation with IL-2 + IL-12 (Figure 5A, bottom right panel). The surface expression of DNAM1 was lower in pNK cells stimulated with IL-2 + IL-12 when compared to cells treated with IL-2, with Dex increasing the level of expression of DNAM1 in cells with IL-2 + IL-12 (Figure 5B). Evaluation of the surface expression of DNAM1 displayed a significant increase in the DNAM1^dim^ subset and a decrease in DNAM1^bright^ subset in pNK cells stimulated with IL-2 + IL-12 (Figure 5B). Dex also increased expression of both CD94 and NKG2A in cells stimulated with IL-2 and IL-2 + IL-12 (Figures 5C,D). Thus, Dex increases the percentage of IL-2 + IL-12 stimulated pNK cells positive for CD16 while augmenting the surface expression of both CD94 and NKG2A and elevating the level of expression of DNAM1 in pNK cells stimulated with IL-2 + IL-12.

### Dexamethasone Augments Secondary Recall Responses by NK Cells *In Vitro*

**Figure 6:**
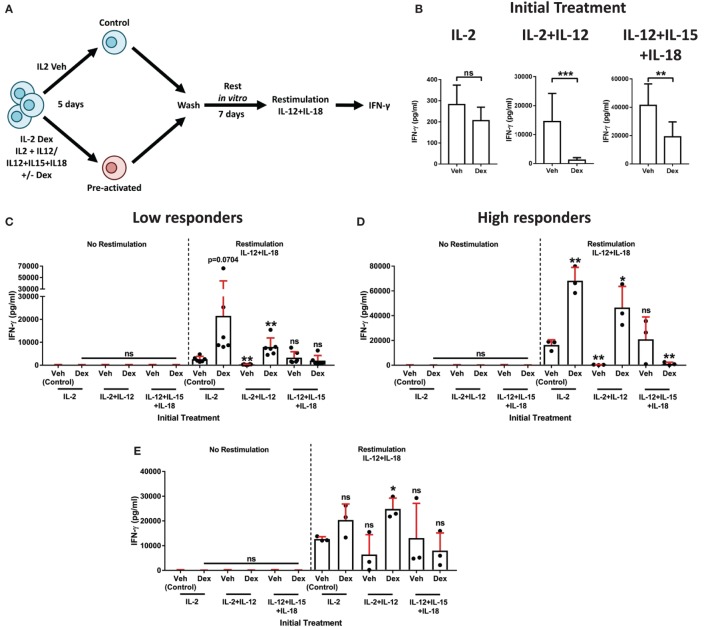
**Dexamethasone augments natural killer (NK) cell secondary recall responses**. **(A)** Schematic of the experimental approach for the preactivation of primary human NK (pNK) cells, then restimulation with interleukin-12 (IL-12) + interleukin-18 (IL-18). **(B–E)** pNK cells were initially treated, first with DMSO, as a vehicle control, or Dex (100 nM) for 1 h, then stimulated with interleukin-2 (IL-2) (200 U/ml), IL-2 (200 U/ml) + IL-12 (10 ng/ml), or IL-12 (10 ng/ml) + interleukin-15 (IL-15) (5 ng/ml) + IL-18 (100 ng/ml) for 5 days. Cells were then washed three times and cultured for 7 days with IL-2 (200 U/ml) to support survival. After 7 days, cells were washed and, either, left without restimulation (no restimulation; IL-2, 200 U/ml) or restimulated with IL-12 (10 ng/ml) + IL-18 (100 ng/ml), for 18 h. **(B)** Following the initial treatment, cell supernatant was assessed for interferon-γ (IFN-γ) production by enzyme-linked immunosorbent assay (ELISA) to confirm cellular activation. Data (mean ± SD) depict nine independent donors. Samples are compared by unpaired, two-tailed Student’s *t*-test (**p < 0.005; ***p < 0.0005; ns, not significant). **(C–E)** After restimulation with IL-12 + IL-18 for 18 h, IFN-γ production was assessed by ELISA. IFN-γ release was determined for **(C,D)** the entire population of cells present following the 7-day rest period, or **(E)** pNK cells were resuspended at equal densities for each initial treatment prior to restimulation. Due to donor variation, the data were separated into **(C)** low (<5,000 pg/ml) and **(D)** high (>10,000 pg/ml) responding donors upon restimulation, according to the IL-2 vehicle control. Graphs (mean ± SD) show data from **(C)** six, **(D)** three, or **(E)** three independent donors. **(C–E)** Samples are compared to the relevant IL-2 vehicle control by unpaired, two-tailed Student’s *t*-test (*p < 0.05; **p < 0.005; ns, not significant).

In addition to having established links with pNK cell activation and IFN-γ production, DNAM1, CD94, and NKG2A have, more recently, been associated with memory and memory-like NK cells (19, 42). Such characteristics have been defined by the presence of an enhanced secondary recall response, following a rest period after the resolution of an initial response (14–16). Therefore, we hypothesized a dichotomy of GC action on IL-2 + IL-12 stimulated pNK cells, initially suppressing IFN-γ production, but also priming these cells for an enhanced response upon restimulation. To test this, we adapted an experimental approach previously used to identify memory-like pNK cells (19). pNK cells were treated with Dex or a vehicle control for 1 h, then initially stimulated the cells for 5 days with either IL-2 + IL-12 or IL-12 + IL-15 + IL-18, using IL-2 alone as a control. This was followed with a 7-day rest period *in vitro* with IL-2 to support survival. Cells were subsequently restimulated with IL-12 + IL-18, or with IL-2 as a control (Figure 6A). The initial stimulation with IL-2 + IL-12 or IL-12 + IL-15 + IL-18 triggered the secretion of IFN-γ, an effect that Dex potently suppressed (Figure 6B).

To determine whether Dex induced an enhanced secondary recall response in pNK cells, we compared IFN-γ secretion after the second stimulation (as outlined in schematic of the experimental approach, Figure 6A). In a first version of this experiment, all viable cells following a 7-day rest period were used to establish the secondary response (Figures 6C,D). Due to donor variability, individuals were grouped according to the responsiveness of the IL-2 control group following restimulation (low, <5,000 pg/ml, Figure 6C; high, >10,000 pg/ml, Figure 6D). As expected, IFN-γ secretion was negligible in the absence of restimulation (no restimulation, Figures 6C,D). However, the addition of IL-12 + IL-18 elicited induction of IFN-γ protein (comparison of the restimulation and no restimulation control groups; Figures 6C,D).

Primary human NK cells initially treated with Dex in combination with either IL-2 or IL-2 + IL-12 displayed an enhanced production of IFN-γ following restimulation. For IL-2 + IL-12 + Dex specifically, the observed increase in IFN-γ was statistically significant in both the low and high responding donors (Figures 6C,D). By contrast, initial treatment with IL-2 + IL-12 without Dex resulted in a dramatic reduction of IFN-γ secretion after restimulation, while initially treating cells with IL-12 + IL-15 + IL-18 had a negligible effect on the production of IFN-γ following restimulation (Figures 6C,D). The inclusion of Dex when initially treating pNK cells with IL-12 + IL-5 + IL-18 had no effect on IFN-γ production in those donors that exhibited a low response (Figure 6C). Whereas, in the individuals that displayed an elevated response to stimulation with IL-12 + IL-18, preactivation with IL-12 + IL-15 + IL-18 Dex resulted in a significantly reduced secondary response, when compared to the restimulated control group (Figure 6D). Overall, these data establish that initial exposure to Dex in combination with IL-2 + IL-12 elicits a heightened secondary recall response in pNK cells *in vitro*.

Augmented secondary responses could result from the expansion in cell numbers. Thus, in a second version of this experiment, we set out to establish whether GCs augment pNK cell reactivity directly, not just *via* promoting their expansion and survival. For this, following preactivation and a 7-day rest period *in vitro* (Figure 6A), pNK cells were resuspended at matched densities prior to restimulation (Figure 6E). Again, IFN-γ release was minimal without restimulation (No restimulation, Figure 6E), while the production of IFN-γ was triggered by IL-12 + IL-18 (Restimulation, Figure 6E). As observed above, initial treatment with IL-2 + IL-12 Dex promoted an enhanced production of IFN-γ upon restimulation when compared to the restimulated control group (Figure 6E). Taken together, these results establish that GCs augment both the expansion and reactivity of pNK cells to elicit an enhanced secondary recall response. Overall, these data display a dichotomy of GC action on pNK cell stimulated with IL-2 + IL-12: initially suppressing the immune response, but paradoxically enhancing cell survival, proliferation, and reactivity. Pre-exposure to GCs in combination with IL-2 + IL-12 subsequently primes pNK cells for an enhanced recall immune response.

## Discussion

Due to their anti-inflammatory, pro-apoptotic, and antiemetic properties, GCs have been widely used in the treatment of inflammatory disorders and cancer. For example, methylprednisolone (27) and shown here, Dex, inhibits IL-2- or IL-15-mediated proliferation of NK cells. However, GCs have also been reported to enhance cell proliferation and survival (32). Hydrocortisone has been described to increase the proliferation and survival of CD56^+^ cells when cultured with either IL-2 or IL-15, leading to the enhanced expansion of NK cells (32). Further clarifying the effects of GCs on NK cell functions could have important consequences in the way cancer and inflammatory disorders are managed clinically. Here, we establish a clear dichotomy in the action of Dex, initially suppressing NK cell activity while, dependent on the local cytokine milieu, conferring an enhanced functional response after restimulation.

Previous research has shown that another clinically important corticosteroid, methylprednisolone, conferred different effects on NK cells dependent on whether or not the cells were cultured in IL-2 or IL-15 (27). Specifically, NK cell survival was reduced by methylprednisolone for cells cultured in IL-2 but not IL-15. By contrast, we found that NK cell survival was worsened by Dex for cells cultured in IL-15, not IL-2. Thus, different GCs may regulate NK cell expansion and survival differently. To test this possibility directly, further work is required using equipotent doses of different GR ligands and matched NK cell culture conditions.

Our data show that Dex augments the proliferation of both fresh and *in vitro*-cultured NK cells stimulated with IL-2 + IL-12 while protecting these cells from cytokine-induced cell death. Most importantly, these cells also displayed an enhanced secondary recall response. Thus, pretreatment with a combination of Dex and cytokines may prove an important component of a successful adoptive transfer immunotherapy protocol. The infusion of NK cells (or NK cell derivatives, such as CAR NK cells) into cancer patients may be an effective therapy (43–45) but is hampered by insufficient expansion, short term survival and transient cytolytic activity. If the therapeutic potential of NK cell-based immunotherapy is to be realized, the development of activation strategies that result in the *in vivo* expansion of long-lived tumor-reactive NK cells is required.

The mechanism by which Dex can augment the proliferation, survival, and reactivity of human NK cells stimulated by IL-2 + IL-12 is not obvious. It has long been known that cytokines can induce apoptosis in NK cells and that this is at least in part caused by NK cell production of TNF-α (46). Thus, it is possible that Dex is able to increase NK cell survival by reducing the production of TNF-α. However, Dex appears to augment NK cell survival only in specific cytokine milieu, while TNF-α transcription is inhibited more generally, implying that additional effects must occur. In T cells, Dex has been shown to inhibit STAT4 phosphorylation induced by IL-12 (47). STAT4 is important for NK cell proliferation and the generation of virus-specific memory NK cells (48) although the role of STAT4 phosphorylation specifically has not been established. It is possible that the mechanism by which Dex can augment the proliferation, survival, and reactivity of human NK cells is *via* effects on STAT4-mediated signaling.

Natural killer cell memory can be defined by an enhanced secondary recall response, following a rest period after the resolution of an initial response (14–16) and, although we have not used protocols identical to those used previously for the identification of memory-like properties in NK cells, our findings indicate that it is interesting to assess whether or not dexamethasone impacts the generation of memory or memory-like NK cells. More broadly, an immunosuppressive environment, such as that triggered by GCs, could be an important factor in the generation of memory NK cells.

## Ethics Statement

The use of human blood was approved by our University Research Ethics Committee (05/Q0401/108).

## Author Contributions

DM and DD conceived the project, designed experiments, and wrote the manuscript; DM performed experiments and analyzed the data.

## Conflict of Interest Statement

The authors declare that the research was conducted in the absence of any commercial or financial relationships that could be construed as a potential conflict of interest. The reviewer, AL, and handling editor declared their shared affiliation, and the handling editor states that the process nevertheless met the standards of a fair and objective review.
