# Supplementary material for: Distinct Effects of Dexamethasone on Human Natural Killer Cell Responses Dependent on Cytokines
Source: Front Immunol. 2017 Apr 13;8:432. doi: 10.3389/fimmu.2017.00432 (PMC5389971; doi:10.3389/fimmu.2017.00432)
Supplement: Supplementary file 1 [file Presentation_1.pdf]

Supplemental Figures

Figure S1.

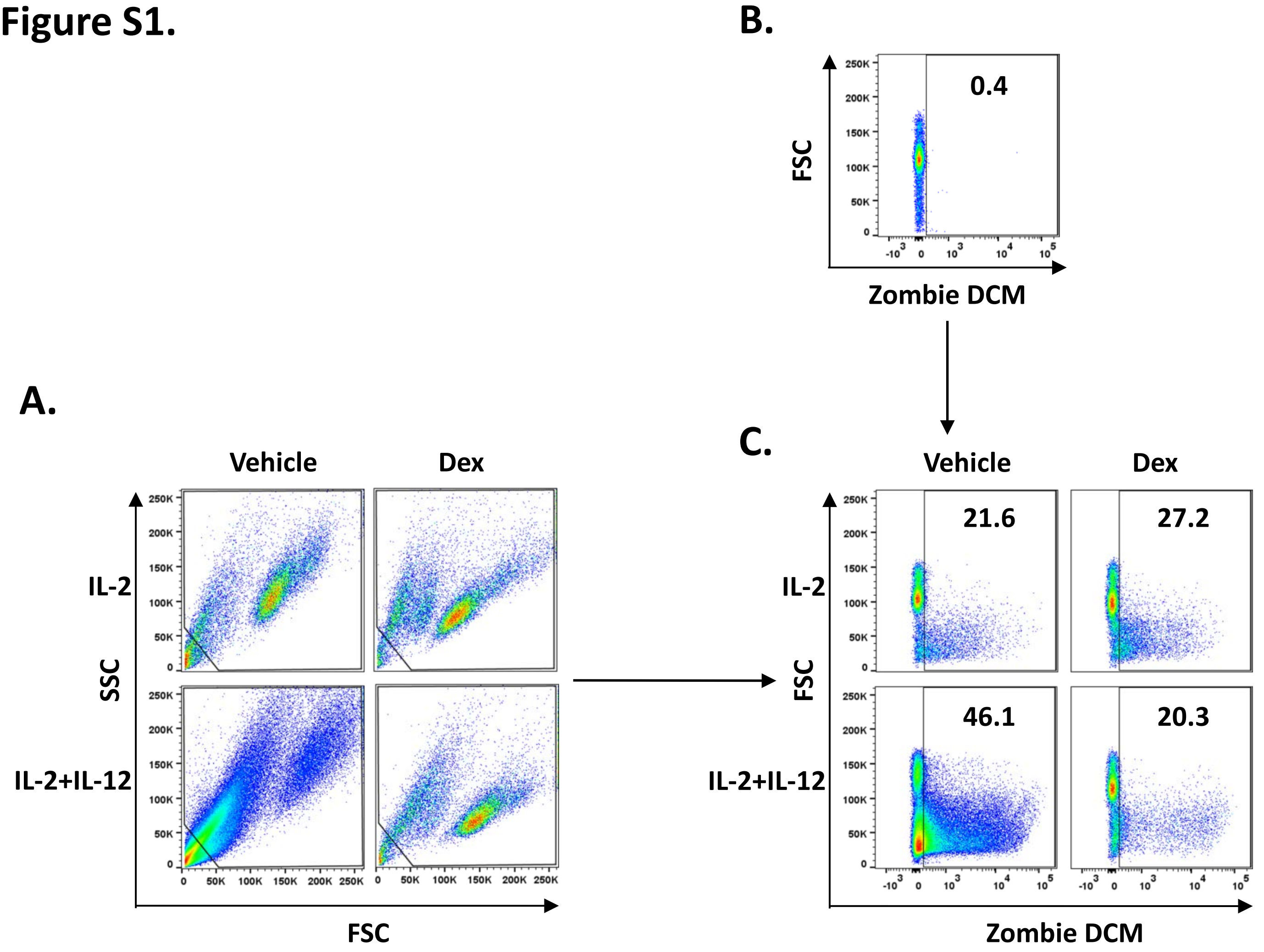

Figure S1. Flow cytometry gating strategy used to determine cell death in Figures 2 and S2. Gating strategy is displayed using the data in Figure 2C and H. (A) Initially cell debris was removed using gating based on side scatter against forward scatter. Based on an unstained control sample (B), the resulting populations from panel (A) were gated on Forward Scatter against Live/Dead stain, Zombie Dead Cell Marker (DCM), to determine cell death (C). The number of events in the IL-2+IL-12 vehicle condition in panel (A) is greater than other conditions as data was acquired to equalise the number of live dividing cells.

Figure S2.

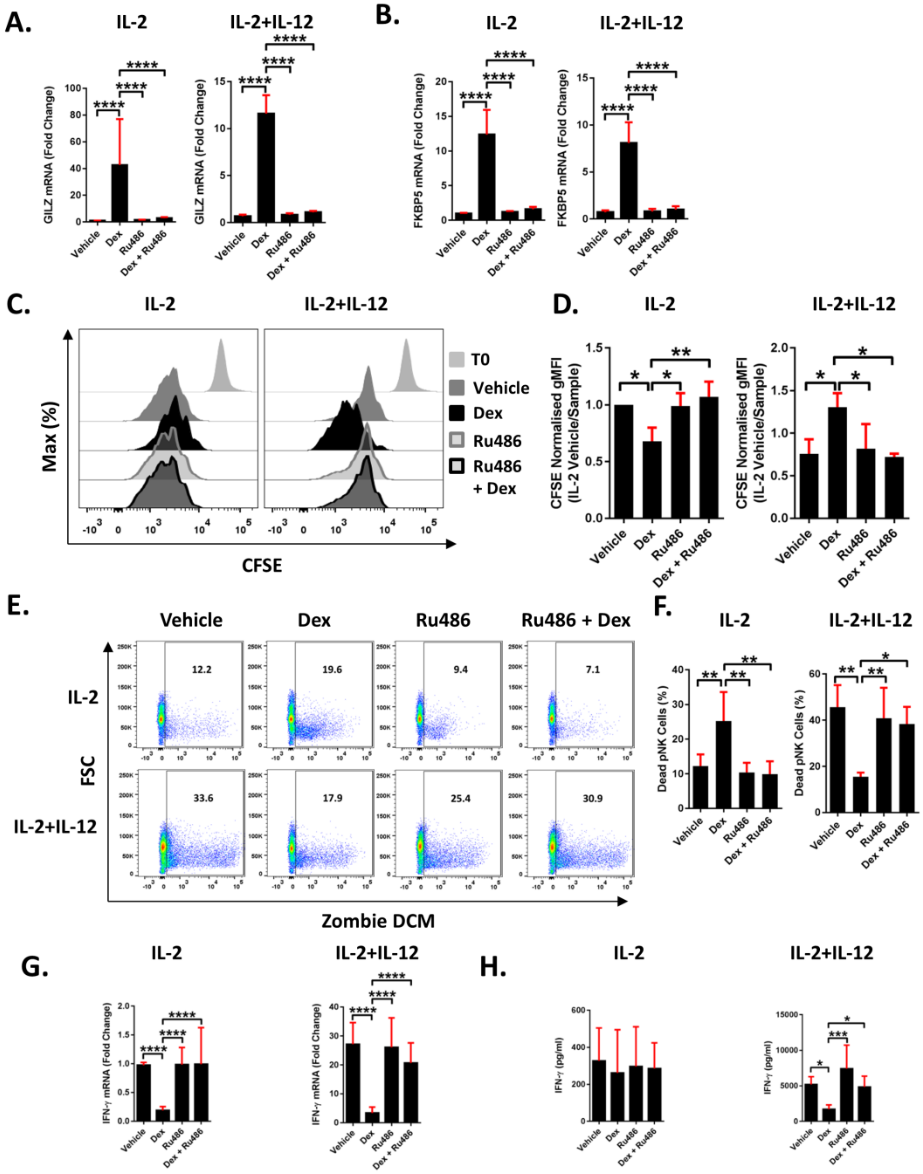

Figure S2. Enhanced proliferation by GCs and the immunosuppressive effect is dependent on GR. (A-B) Following treatment with vehicle, Dex (100nM) and Ru486 (1µM), as indicated, for 1 h, pNK cells were incubated with either IL-2 (200 U/ml) or IL-2 (200 U/ml) + IL-12 (10ng/ml) for 18 h. Expression of (A) GILZ and (B) FKBP5 mRNA was analysed by RT-qPCR. Data is normalised to GAPDH and displayed as a fold change over the IL-2 vehicle control. Graphs (mean ±SD) combine data for five independent experiments. (C-F) pNK cells were first labelled with CFSE (0.5µM) and then treated with a vehicle control, Dex (100nM) and Ru486 (1µM), as indicated, for 1 h. Pre-treated cells were cultured for 5 d at 37°C with either IL-2 (200 U/ml) or IL-2 (200 U/ml) + IL-12 (10ng/ml). (C-D) Proliferation and (E-F) cell survival was assessed by flow cytometry. Representative (C) histograms (gated on live $CD56^{+}$pNK cells) and (E) percentages of dead pNK cells are shown. Graphs (mean ±SD) show the quantification of (D) CFSE gMFI (normalised to IL-2 vehicle) for 3 (IL-2) or 4 (IL-2+IL-12) independent donors, and (F) percentage of dead pNK cells, from 5 (IL-2) or 4 (IL-2+IL-12) independent experiments. (G-H) pNK cells were treated as described in (A-B). (G) Expression of IFN-γ mRNA was analysed by RT-qPCR. Data is normalised to GAPDH and displayed as a fold change over the IL-2 vehicle control (mean ±SD), for 5 (IL-2) or 4 (IL-2+IL-12) independent donors. (H) IFN-γ release was measured by ELISA. Graphs (mean ±SD) combine data for three independent experiments. Samples are compared by one-way ANOVA (*p<0.05; **p<0.005, ***p<0.0005, ****p<0.0001). For simplicity only significant results are labelled.

Figure S3.

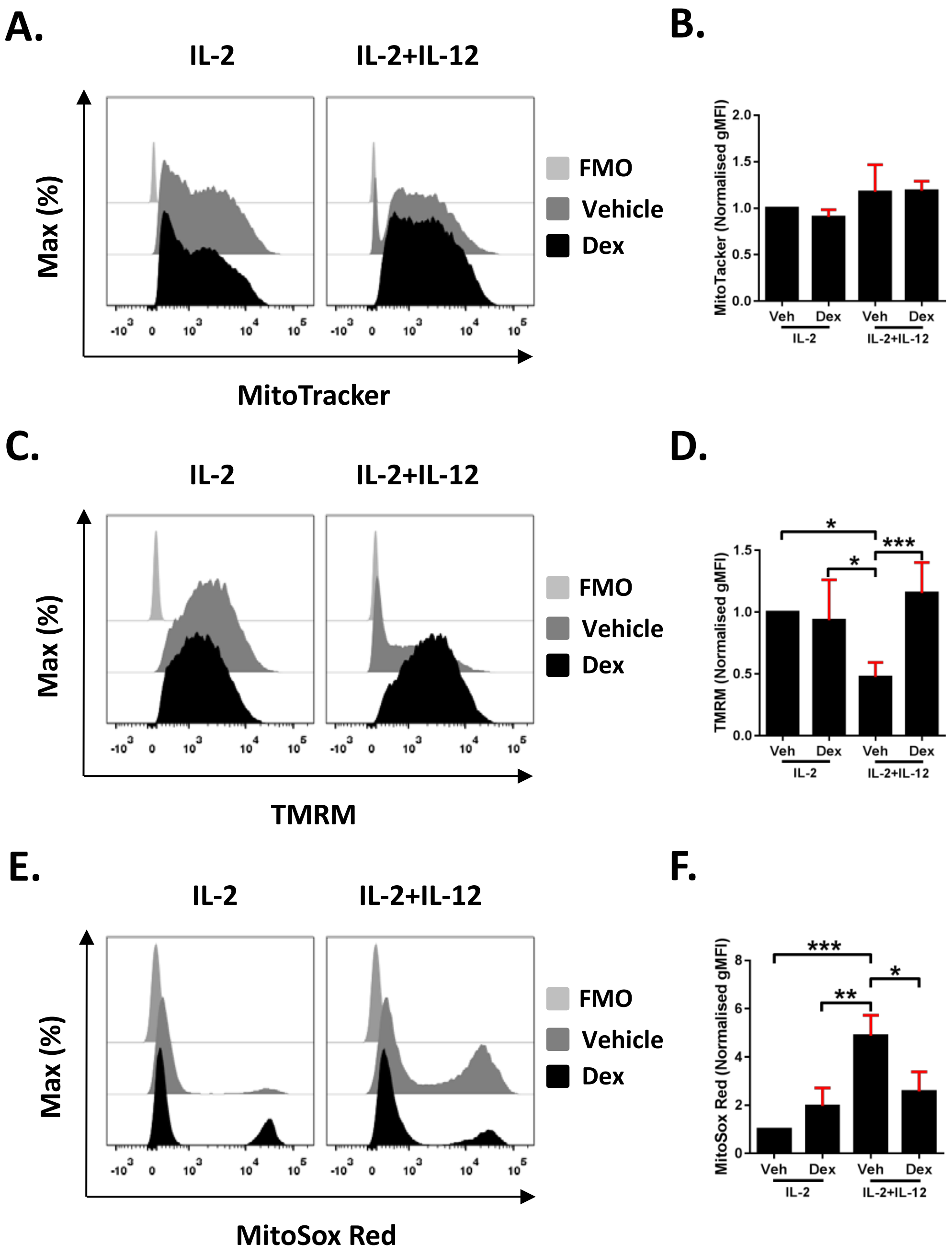

Figure S3. GCs restore mitochondrial function. pNK cells, were treated with Dex (100nM) for 1 h, then cultured for 5 d at 37°C with either IL-2 (200 U/ml) or IL-2 (200 U/ml) + IL-12 (10ng/ml). Shown are representative histograms and quantification of gMFI, normalised to cells treated with IL-2 plus DMSO (vehicle control), for (A-B) MitoTracker Green staining, (C-D) Tetramethylrhodamine methyl ester (TMRM) staining or (E-F) MitoSox Red staining, for the indicated treatments. Graphs (mean ±SD) are representative of (B) 4, (D) 4, or (F) 3 independent experiments. Samples are compared by one-way ANOVA (*$p<0.05$; **$p<0.005$, ***$p<0.0005$). For simplicity only significant results are labelled. FMO, Fluorescence Minus One.

Figure S4.

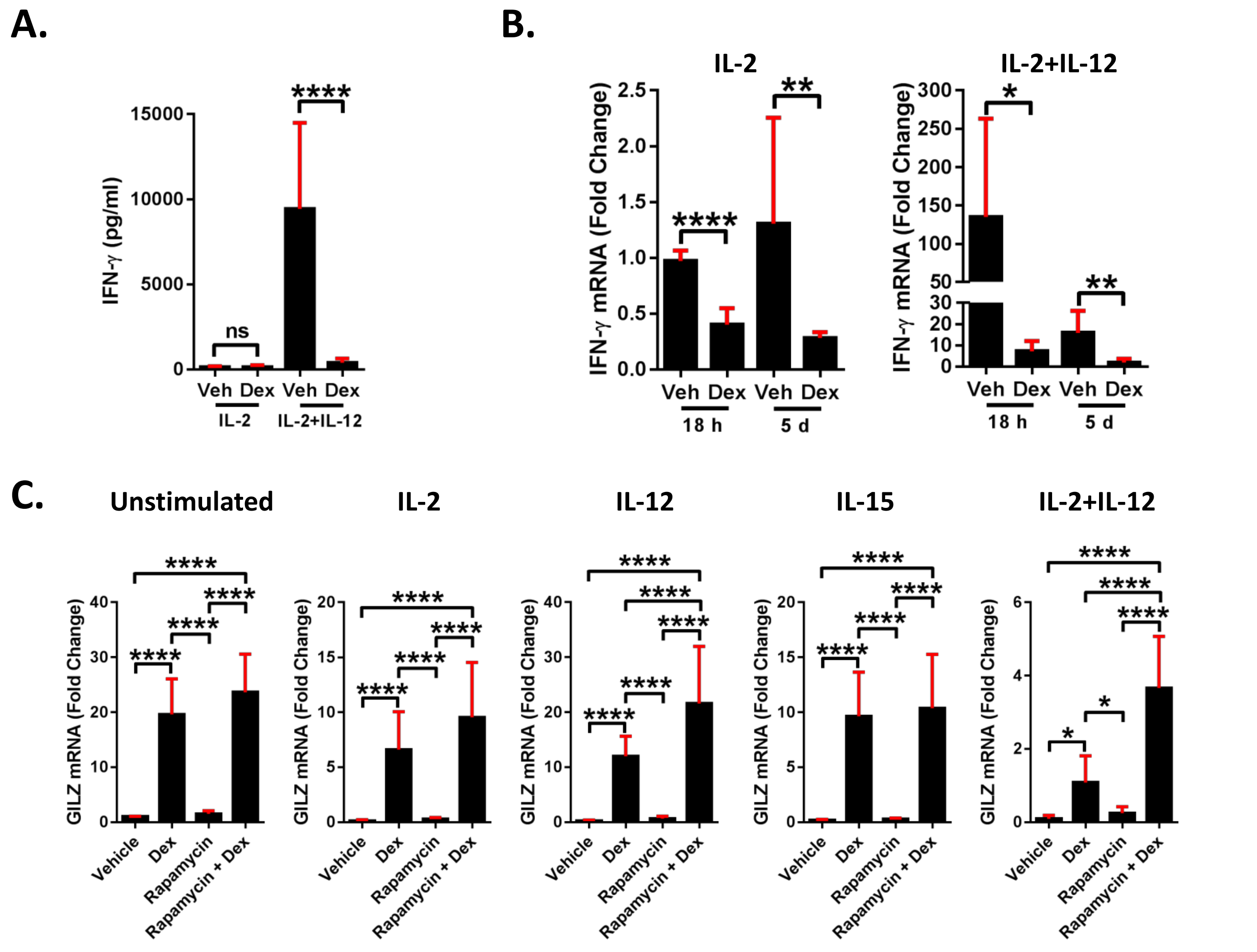

Figure S4. GCs suppress production of IFN-γ throughout culture and Rapamycin enhances GC-mediated induction of GILZ. (A-B) pNK cells were treated with a vehicle control or Dex (100nM) for 1 h, then cultured with IL-2 (200 U/ml) or IL-2 (200 U/ml) + IL-12 (10ng/ml) for 18 h or 5 d. (A) IFN-γ release after 5 d was measured by ELISA. Graphs (mean ±SD) combine data from 4 independent experiments. (B) Expression of IFN-γ mRNA was assessed by RT-qPCR and normalised to GAPDH. Graphs (mean ±SD) show the fold change over the 18 h IL-2 vehicle control, for three independent experiments. Samples are compared by one-way ANOVA (*$p<0.05$; **$p<0.005$, ****$p<0.0001$). (C) pNK cells were pre-treated with a vehicle control or Dex (100nM) for 1 h, and then treated with Rapamycin (10nM), as indicated. pNK cells remained either unstimulated or stimulated with IL-2 (200 U/ml), IL-12 (10ng/ml), IL-15 (5ng/ml) or IL-2+IL-12 for 18 h. The production of GILZ mRNA was analysed by RT-qPCR. Data is normalised to GAPDH and displayed as a fold change over the unstimulated vehicle control. Graphs (mean ±SD) combine data for 4 (IL-15) or 5 (all other conditions) independent experiments. Samples are compared by one-way ANOVA (*$p<0.05$; **$p<0.005$, ***$p<0.0005$, ****$p<0.0001$). For simplicity only significant results are labelled.

Figure S5.

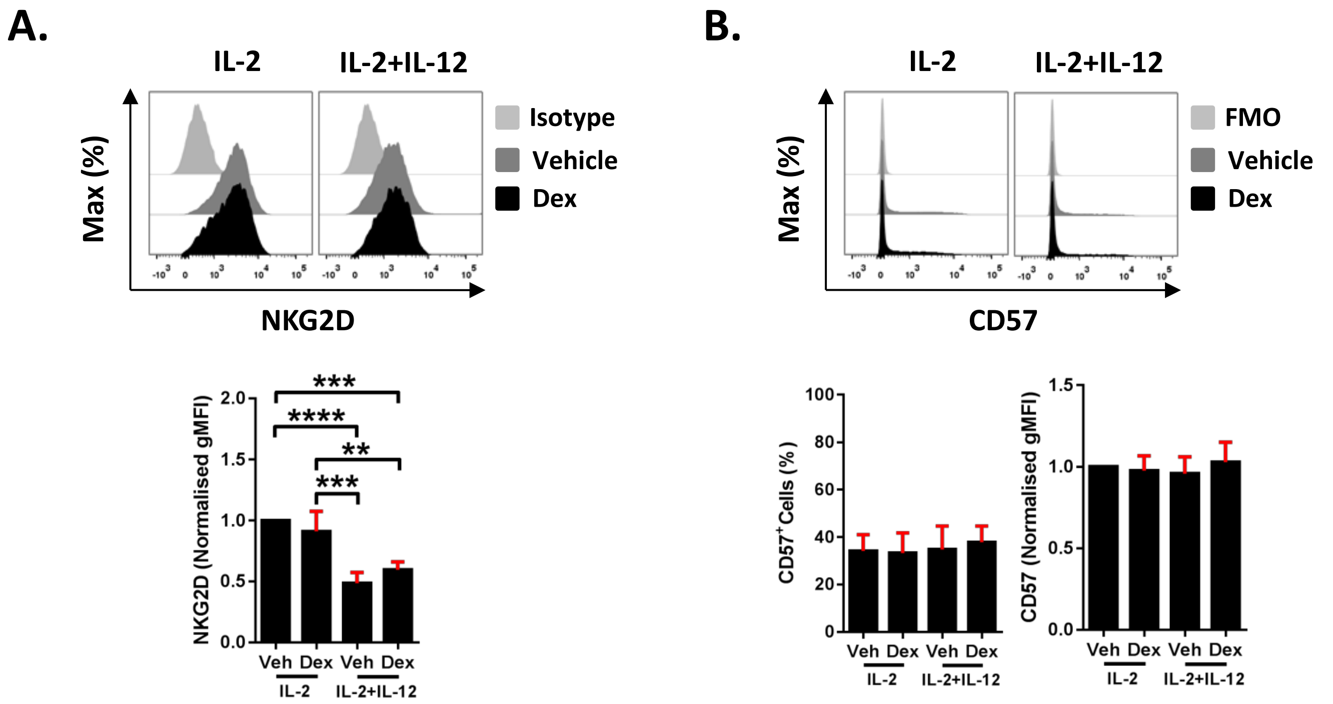

Figure S5. Dexamethasone has no effect on the expression of NKG2D and CD57. pNK cells pre-treated with Dex (100nM) for 1 h, were cultured for 5 d with either IL-2 (200 U/ml) or IL-2 (200 U/ml) +IL-12 (10ng/ml). Cells were assessed for (A) NKG2D and (B) CD57 by flow cytometry. Representative histograms are shown. Graphs depict the gMFI, normalised to IL-2 vehicle, for (A) NKG2D and the percentage of cells positive for (B) CD57, and the gMFI of the positive populations, normalised to IL-2 vehicle. Data (mean ±SD) combines four independent experiments. Samples are compared by one-way ANOVA (**$p<0.005$; ***$p<0.0005$; ****$p<0.0001$). For simplicity only significant results are labelled. FMO, Fluorescence Minus One.
